# PRF Lysates Enhance the Proliferation and Migration of Oral Squamous Carcinoma Cell Lines

**DOI:** 10.3390/dj11100242

**Published:** 2023-10-19

**Authors:** Layla Panahipour, Rebecca Croci, Sara Guarnieri, Reinhard Gruber

**Affiliations:** 1 Department of Oral Biology, University Clinic of Dentistry, Medical University of Vienna, 1090 Vienna, Austria; layla.panahipour@meduniwien.ac.at (L.P.); rebecca.croci@studenti.unipr.it (R.C.); sara.guarnieri@studenti.unipr.it (S.G.); 2 Department of Periodontology, School of Dental Medicine, University of Bern, 3010 Bern, Switzerland; 3 Austrian Cluster for Tissue Regeneration, 1200 Vienna, Austria

**Keywords:** platelet-rich fibrin, oral epithelial cells, oral squamous carcinoma cells, proliferation, migration

## Abstract

Platelet-rich fibrin (PRF) is an autologous fibrin-rich matrix where activated platelets and leucocytes accumulate. PRF has a wide spectrum of clinical indications with the overall aim of supporting tissue regeneration which in dentistry includes the healing of healthy oral mucosa with epithelial cells. In oral squamous cell carcinoma lesions, however, epithelial cells undergo malignant transformation, indicated by their unrestricted proliferation and migration potential, which should not be further enhanced by a wound-healing formula. Yet, little is known about how oral squamous cell carcinomas respond to PRF lysates. The aim of the present study was, therefore, to test the capacity of PRF lysates to change the transcriptome of HSC2 oral squamous carcinoma cells and perform bioassays to support the findings. Based on the RNAseq analysis, PRF lysates caused an increase in the genes functionally linked to cell replication and migration. In support of this screening approach, PRF lysates enhanced the proliferation of HSC2 oral squamous carcinoma cells, as indicated by ^3^[H]-thymidine incorporation, cell counting, and the expression of proliferation-related genes. Moreover, PRF lysates sped up cell migration in a scratch assay requiring actin polymerization. Taken together, our data showing that PRF lysates are mitogenic and stimulate motility of oral squamous carcinoma cell lines could be an indication that treatment with PRF in cases of oral carcinoma should be carefully considered.

## 1. Introduction

Platelet-rich fibrin is a blood-derived preparation of coagulated blood after centrifugation [1]. Blood is harvested by venous puncture and immediately centrifuged so that coagulation takes place after the separation of the plasma fraction from the bulk of the erythrocytes. The plasma fraction containing the platelets and some leukocytes then undergoes coagulation and can be separated from the red clot. To produce PRF membranes, the plasma clot is squeezed between two metal plates prior to its clinical use, for instance, to fill extraction sockets [2,3] and to prepare PRF blocks for bone augmentation [4]. Recent reviews provide an overview of PRF uses in dentistry, including the treatment of periodontal intrabony defects [5] and gingival recessions [6]. Under these conditions, PRF, similar to a blood clot, provides a surface for epithelial cells to grow from the edges toward the center of the defect [7]. Re-epithelialization of the oral mucosa is controlled by growth factors [8] and clinical evidence supports that PRF helps the re-epithelialization of the healthy oral mucosa [9].

Skin wound healing research underpins the impact of platelet-derived growth factors and other bioactive molecules on dermal keratinocytes; for instance, platelet-derived concentrates stimulated keratinocyte proliferation in vitro [10], and human platelet lysate enhanced the migration and proliferation of keratinocytes [11]. Other findings, however, suggest that platelet-released growth factors and platelet-rich plasma can inhibit the proliferation of keratinocytes [12,13]. In the context of dentistry, platelet-rich plasma was seen to enhance the proliferation of SCC25 epithelial-like cells originally isolated from the tongue of a patient with squamous cell carcinoma [14]; however, these cells are transformed cell lines representing an oral pathology. This study is an indication of the tumor-supporting action of platelet concentrates and, thus, metastatic potential.

One can imagine an in vivo scenario when the subject has a clinically undetectable oral tumor or a premalignant lesion [15] where the use of PRF might initiate a full-blown oral squamous cell carcinoma (OSCC), an entity that accounts for about 92–95% of all cancer in the oral cavity [16]. It takes years for OSCC to progress, and risk factors including smoking, alcohol, and human papillomavirus (HPV) infection are obvious [17,18]. Other related risk factors are the precancerous conditions of oral leukoplakia [19] and oral lichen planus [20]. Presumably, platelets also play a role in OSCC pathology. Platelet microthrombi were detected in OSCC and linked to lymph node metastasis [21]. Overall, platelets enhance the invasiveness of human and murine cancer cell lines [22,23] and platelet injection promotes the formation of metastatic nodules in murine cancer metastasis models [24]. In general, platelets alter the epithelial tumor cell attributes to drive metastasis [25].

In vitro, platelet–tumor interactions have been studied with Ca9.22 and HSC3 cells originating from OSCC [26]. We have previously implemented our established oral squamous carcinoma cell line HSC2 as a bioassay for testing the responsiveness to lysates prepared from PRF membranes, however, in the context of inflammation research [27,28]. Today’s attempts to assess the response of HSC2 and other cell types to PRF lysates are based on RNA sequencing, an omics technology that provides insight into how the genetic signature of cells changes [29]. Similar to the understanding of the response of gingival fibroblasts to milk, it is now time to identify how HSC2 cells respond to PRF lysates based on RNAseq, followed by an analysis of genes regulating the cell cycle such as cyclin D1, Ki67, and PCNA [30]. Also, histones providing structural support for chromosomal DNA are required when cells duplicate and are considered proliferation markers [31]. Heterochromatin involving the histones is also required for cell migration [32]. In addition, we use TR146 cells originating from the human neck metastasis of a buccal carcinoma and primary oral epithelial cells as a control. Thus, the primary aim of the study is to understand the overall response of HSC2 oral squamous carcinoma cells to PRF lysates and perform the respective proliferation and migration bioassays, to understand if PRF potentially supports the malignancy of oral squamous carcinoma cells.

## 2. Material and Methods

### 2.1. Oral Squamous Cell Carcinoma Cell Lines HSC2, TR146, and Primary Cells

HSC2 and TR146 are cell lines originating from oral squamous cell carcinomas (Health Science Research Resources Bank, Sennan, Japan, and European Collection of Authenticated Cell Cultures, respectively). Both cell lines were grown in DMEM serum-containing medium and antibiotic-antimycotic solution (Sigma-Aldrich, St. Louis, MO, USA). Primary human oral epithelial cells were isolated from gingiva obtained during wisdom tooth removal from healthy donors who gave their informed consent (Ethical Committee of the Medical University of Vienna, Nr. 631/2007). Epithelial cells were seeded at 2.5 × 10^5^ cells/cm^2^ onto culture dishes one day prior to overnight exposure to 30% PRF lysates in serum-free media.

### 2.2. PRF Lysates, Buffy Coat, and Platelet-Poor Plasma

The preparation of PRF was approved by the Ethical Committee of the Medical University of Vienna (Nr. 1644/2018). Glass tubes (Bio-PRF, Venice, FL, USA) supporting blood coagulation were subjected to 700 g for 8 min (Z306 Hermle, Universal Centrifuge, Wehingen, Germany). The yellow PRF clot was removed and squeezed between layers of gauze. One cm of PRF membrane was used per mL of serum-free medium. Liquid PRF fractions were prepared with polyethylenterephthalat tubes (Greiner Bio-One GmbH, Kremsmünster, Austria) after blood centrifugation at 2000× *g* for 8 min (Z306 Hermle). The uppermost 2 mL platelet-poor plasma (PPP) and the 1 mL buffy coat (BC) were then collected. All preparations underwent two cycles of freeze–thawing at −80 °C, followed by 30 s sonication at room temperature (Sonopuls 2000.2, Bandelin electronic, Berlin, Germany). The lysates underwent centrifugation at 15,000× *g* for 10 min before 1 mL aliquots of the supernatant were stored frozen for less than one month.

### 2.3. RNA Sequencing

Following RNA extraction (ExtractMe, Blirt S.A., Gdańsk, Poland), the quality was assessed with an Agilent 2100 Bioanalyzer (Agilent Technologies, Santa Clara, CA, USA). Sequencing libraries were prepared at the Core Facility Genomics at the Medical University of Vienna using the NEBNext Poly (A) mRNA Magnetic Isolation Module and the NEBNext Ultra™ II Directional RNA Library Prep Kit for Illumina, according to manufacturer’s protocols (New England Biolabs, Ipswich, MA, USA). Libraries were QC checked on a Bioanalyzer 2100 (Agilent Technologies) using a High Sensitivity DNA Kit and quantified using a Qubit dsDNA HS Assay (Invitrogen, Waltham, MA, USA). Pooled libraries were sequenced on a NextSeq500 device (Illumina, San Diego, CA, USA) with a 1 × 75 bp single-end sequencing method. Roughly 25 million reads were created per sample. Reads in fastq format were aligned to the human reference genome version GRCh38 (www.ncbi.nlm.nih.gov/grc/human, accessed on 25 January 2022) with Gencode 29 annotations (www.gencodegenes.org/human/release_29.html, accessed on 25 January 2022) using the STAR aligner 55 version 2.6.1a in the two-pass mode. Reads per gene were counted by STAR and differential gene expression was calculated using DESeq2 56 version 1.22.2. DESeq2. The STRING database was used to show protein–protein interactions (string-db.org, accessed on 25 January 2022).

### 2.4. Reverse Transcription Quantitative Real-Time PCR

The total RNA was reverse transcribed (LabQ, Labconsulting, Vienna, Austria) and cDNA was amplified on a CFX Connect™ RealTime PCR Detection System (Bio-Rad Laboratories, Hercules, CA, USA). The transcription levels of target genes indicated in Table 1 were normalized to GAPDH using the ΔΔCt method.

### 2.5. Proliferation Assay

Cells were incubated with 30% PRF lysate or left untreated before being pulse-labeled with ^3^[H]thymidine (0.5 mCi/well, Hartmann Analytic, Braunschweig, Germany) exposure for at least 16 h. The plates then underwent liquid scintillation counting (Packard, Meriden, CT, USA). Thymidine incorporation was indicated as counts per minute. In addition, cells were seeded at 3 × 10^4^ cells/cm^2^ and counted after 72 h of exposure with a phase contrast microscope.

### 2.6. Scratch Assay

Cells seeded at 2.5 × 10^5^ cells/cm^2^ were scratched with a sterile plastic micropipette tip to generate a regular gap width. Cells were washed with serum-free medium to remove debris and exposed overnight to 30% PRF lysate with and without the actin polymerization inhibitor cytochalasin D at 1 µM (Sigma-Aldrich).

### 2.7. Statistical Analysis

Single experiments were repeated at least four times. In the figures, the data points represent independent experiments with blood obtained from independent donors. Ordinary one-way ANOVA and paired *t*-tests were used for statistical analysis (Prism v9, GraphPad Software, La Jolla, CA, USA). *p*-values are indicated in the figures.

## 3. Results

### 3.1. RNAseq Analysis

To understand the overall response of the HSC2 cells exposed to the PRF lysate, an RNAseq analysis was performed, and the resulting data are shown in Table 2. Gene ontology revealed an enrichment of the 48 (>10-fold) upregulated genes related to GO:0006270 DNA replication initiation with 4 of 32 (biological process) and GO:0005149 interleukin-1 receptor binding with 3 of 17 (molecular function). Upregulated genes were also enriched for the GO:0000786 nucleosome with 6 of 106, GO:0032993 protein-DNA complex with 7 of 195, and GO:0000228 nuclear chromosome with 12 of 1256 (cellular component). No enrichment was identified for the 34 (>10-fold) down-regulated genes. Data from RNAseq and GO analysis suggest that the most relevant changes caused by the PRF lysates relate to the proliferation but also the expression of IL1 in HSC2 cells.

### 3.2. RT-PCR Analysis

**Figure 1:**
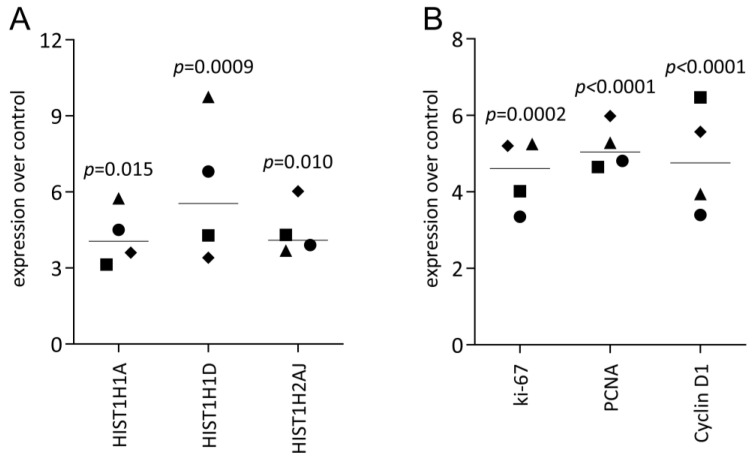
RT-PCR analysis of histones and proliferation markers: HSC2 were exposed to PRF lysates, and the expression changes of histones (**A**) and proliferation marker genes (**B**) identified by RNAseq were evaluated. Data are expressed as the x-fold changes in the PRF-stimulated HSC2 cells compared to the respective untreated controls. Data points represent independent experiments. *p*-values originate from ordinary one-way ANOVA tests.

**Figure 2:**
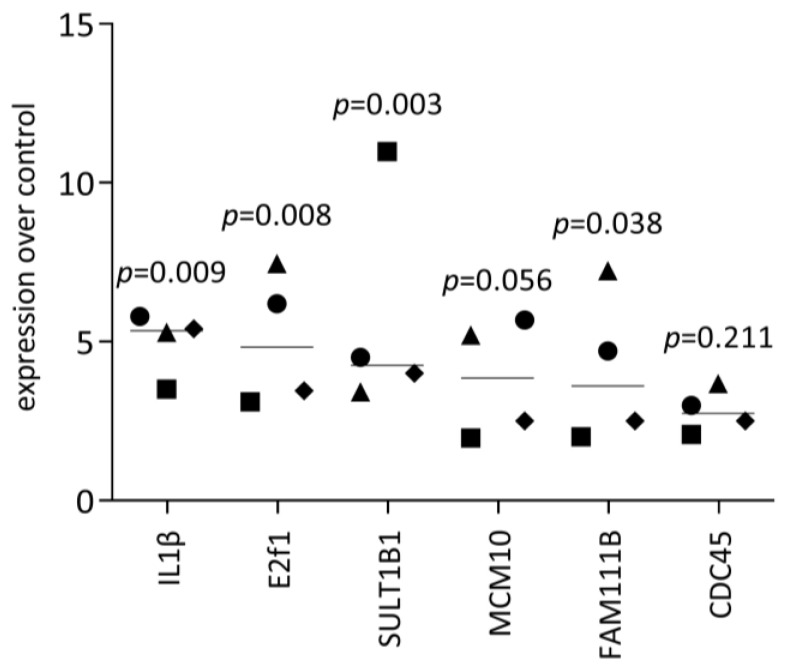
RT-PCR analysis of genes identified by RNAseq: HSC2 were exposed to PRF lysates, and the expression of genes identified by RNAseq was assessed. Data are expressed as the x-fold changes in the PRF-stimulated HSC2 cells over the respective untreated controls. Data points represent independent experiments. *p*-values originate from ordinary one-way ANOVA tests.

To support the observation from RNAseq analysis, we performed an expression analysis, starting with confirming the up-regulation of histone H1-linking DNA and nucleosomes, as well as the core histone HIST1H2AJ (Figure 1A). Consistent with the overall picture obtained from RNAseq, genes that are linked to the cell cycle were increased in the HSC2 cells exposed to PRF lysates, e.g., Ki67, PCNA, and Cyclin D1 (Figure 1B). This was also true for fractions of liquid PRF, namely, buffy coat (BC) and platelet-poor plasma (PPP), as reported in Figure S1. The RNAseq findings are further supported by the increased expression of E2F1 and other genes based on RT-PCR (Figure 2). Finally, we confirmed that PRF lysates, but also BC and PPP, provoke a moderate but significant increase in IL1β expression in HSC2 cells (Figure 2, Figure S2). Taken together, the RT-PCR analysis confirmed the overall impression that PRF lysates exert a mitogenic activity and also enhance IL1 expression in HSC2 cells.

### 3.3. ^3^[H]-Thymidine Incorporations and Cell Counting

**Figure 3:**
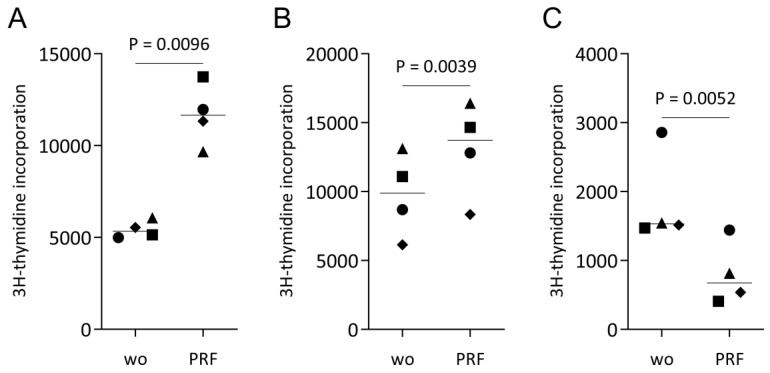
^3^[H]-thymidine incorporations. (**A**) HSC2, (**B**) TR146, and (**C**) primary oral epithelial cells were incubated with and without PRF lysates for 24 h. The cells were pulse-labeled with ^3^[H]-thymidine for the last 6 h of culture and subjected to liquid scintillation counting. Data were expressed as counts. Data points represent independent experiments. *p*-values originate from paired *t*-tests.

**Figure 4:**
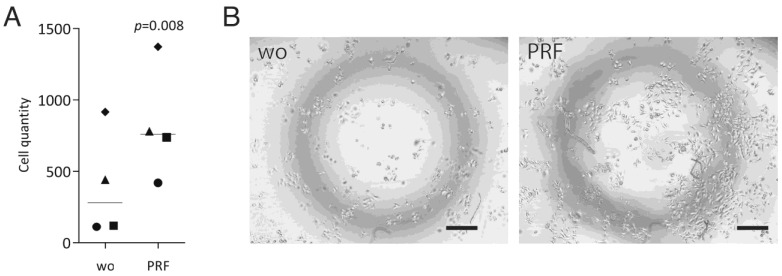
Cell counting: HSC2 cells were incubated with and without PRF lysates for 24 h. Cell counting was performed after 72 h; data are expressed as cells per mL. (**A**) RT-PCR analysis. Data points represent independent experiments. *p*-values originate from paired *t*-tests. (**B**) counted with a phase contrast microscope. The scale bar represents 100 µm.

To support the observation from RNAseq, we performed a ^3^[H]-thymidine incorporation assay and counted the number of HSC2 cells incubated with PRF lysates. When HSC2 cells were exposed to PRF lysates, the incorporation of ^3^[H]-thymidine increased significantly by approximately two-fold (Figure 3A). TR146 also showed a moderate increase (Figure 3B) while, in contrast, the PRF lysates significantly reduced the ^3^[H]-thymidine uptake in primary oral epithelial cells (Figure 3C). This observation was further supported by the counting of HSC2 cells, showing that after 72 h of incubation with lysates from PRF, the cells more than doubled (Figure 4). Further support for the findings comes from data showing that the liquid fractions of blood, namely, the buffy coat and platelet-poor plasma, possess mitogenic activity in HSC2 cells (Figure S3). These findings further support the claim that the PRF lysates exert a mitogenic effect on the transformed cell lines cells, while the opposite was true for the primary oral epithelial cells.

### 3.4. Scratch Assay

**Figure 5:**
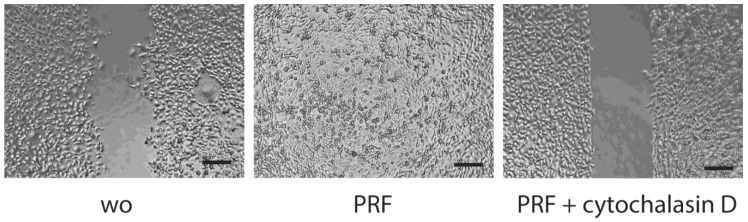
Scratch assay: HSC2 cells grow to confluence before creating a scratch. After removing debris, cells were treated overnight with 30% PRF lysate in the presence or absence of cytochalasin D and inhibition of actin polymerization. wo stands for without and is a serum-free medium. All pictures are taken after 16 h of incubation. The scale bar represents 100 µm.

**Figure 6:**
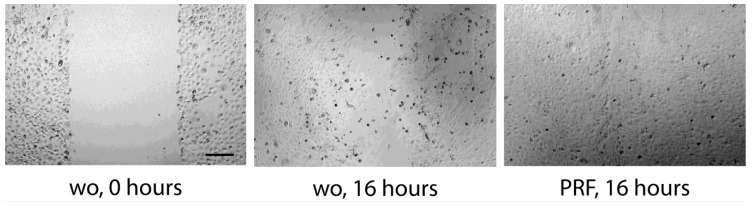
Scratch assay: TR146 cells grow to confluence before creating a scratch. After removing debris, cells were treated overnight with 30% PRF lysate. wo stands for without and is a serum-free medium. Pictures are taken immediately after the scratch (0 h) and after 16 h of incubation. The scale bar represents 100 µm.

**Figure 7:**
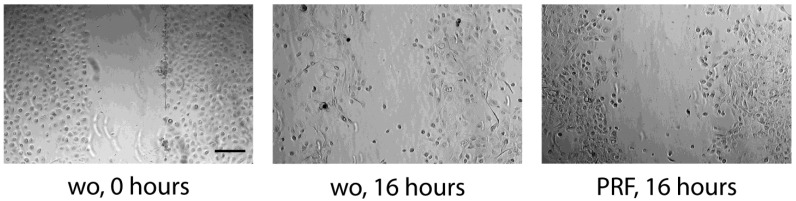
Scratch assay: Primary epithelial cells grow to confluence before creating a scratch. After removing debris, cells were treated overnight with 30% PRF lysate. wo stands for without and is a serum-free medium. Pictures are taken immediately after the scratch (0 h) and after 16 h of incubation. The scale bar represents 100 µm.

To simulate a wound-like situation where the proliferation and migration of epithelial cells are necessary, we performed a classical scratch assay. We can report that the presence of PRF lysates resulted in complete coverage of the injured area, whereas in the presence of serum-free medium, HSC2 failed to fully recover the scratched area (Figure 5). Wound closure was suppressed by inhibition of actin polymerization by cytochalasin D (Figure 5). Consistently, lysates prepared from BC and PPP could support the wound closure of HSC2 scratches. In TR146 cells, wound closure was enhanced by PRF lysates but less impressive (Figure 6). These observations show that PRF lysates support the motility of the two oral squamous carcinoma cell lines. In primary epithelial cells, however, there was no obvious migration support by PRF lysates, but more of a reduced motility of the cells (Figure 7).

## 4. Discussion

PRF applications became a globally recognized therapy to support oral tissue healing, a process that requires oral epithelial cells to proliferate and migrate. However, in the context of oral oncology, platelet-rich plasma was reported to promote the proliferation of squamous cell carcinoma SCC25 cells [14], raising the possibility that PRF might exert a tumor-promoting activity. This assumption is supported by the fundamental understanding that platelets can change epithelial tumor cell characteristics to drive metastasis [25]. Moreover, platelets enhance the invasiveness of cancer cell lines [22,23], platelet injection promotes the in vivo formation of metastatic nodules [24], and platelet aggregates are found in OSCC [21]. This is also the reason why Ca9.22 and HSC3 cells originating from OSCC were used to study platelet–tumor interactions [26]. The present research was based on the fundamental question of how HSC2 cells respond to lysates prepared from PRF membranes on the molecular and cellular level.

Our main finding was based on an RNAseq approach. We identified a genetic signature that highlights two aspects; namely, the increased expression of genes related to cell proliferation and migration apart from the activation of IL1 expression in HSC2 cells. Likewise, the PRF lysates weakly supported TR146 cell proliferation while, in contrast, reducing primary oral epithelial cell proliferation. If we relate our findings to those of others, we have to accept the inconsistency of the present knowledge on how platelet-containing blood fractions modulate the proliferation of epithelial cells; for instance, while platelet-derived concentrates and lysate stimulate keratinocyte proliferation in vitro [10,11], platelet-released growth factors and platelet-rich plasma can also inhibit the proliferation of keratinocytes [12,13]. RNAseq further identified the downregulated genes related to cell replication [33]. In contrast, some found that platelet-rich plasma increased the proliferation of human HaCaT keratinocytes [34], while others found no relevant change in HaCaT proliferation [35]. Considering dentistry, platelet-rich plasma stimulated the proliferation of SCC25 epithelial-like cells originally isolated from the tongue of a patient with squamous cell carcinoma [14], while PRF decreased the viable cell number using an MTT assay of an oral epithelial cell line derived from a gingival carcinoma and metastasis to the cervical lymph node [36]. In light of existing knowledge, our observations provide another piece of information on how PRF membranes differentially affect the proliferation of oral carcinoma and healthy epithelial cells.

Considering the migration of epithelial cells, we have to recognize research on how platelet-containing blood fractions change the healing, and thus the migration, of healthy epithelial cells but also take the oncological properties of OSCC into account. In support of our findings, platelet-rich plasma supported the healing of scratches in HaCaT cells, a spontaneously immortalized keratinocyte cell line from adult human skin [34,37]. This is also true for PRF exosomes enhancing HaCaT cell migration [38] and microvesicles supporting the migration of human keratinocytes [39]. Consistently, PRP improved keratinocyte migration in scratch and transwell migration experiments [40]. PRP further supported the closure of gingival fibroblast scratches in vitro [41]. The proliferation and migration of HaCaT cells might be attributed to PAF (1-O-alkyl-2-acetyl-sn-glycero-3-phosphocholine), a phospholipid-derived regulator released from activated platelets [42]. Interestingly, PAF increased the expression of COX2, a master regulator of inflammatory prostaglandin synthesis [42], supporting our observation that PRF lysates increase IL1 expression in HSC2 cells. IL1 accelerates the migration of normal differentiated airway epithelial cells [43], corneal epithelial cell migration [44], and renal tubular epithelial HK-2 cells [45]—overall suggesting that IL1 might be related to the migration of HSC2 cells.

If we interpret our findings considering the heterogenicity of the current knowledge, we have to restrict our conclusions to the special conditions of our in vitro system and, in particular, state that the HSC2 cells are from oral squamous carcinoma established in the late 1980s [46,47]. The human oral squamous cell carcinoma cells are, therefore, not the oral epithelial cells of a healthy person being treated with PRF membranes. We thus have to be critical and discuss PRF as pro-tumorigenic. Moreover, caution is needed when asserting that wound healing and tumorigenesis have common characteristics, as one is a controlled, regulated physiological process while the other lacks these characteristics. The interpretation of our findings must be further expanded towards oncology, where oral mucositis in the context of radiation and chemotherapy is a heavy burden for patients and PRF might support wound healing under these conditions [48]. In addition, PRF might target oral epithelial cells in precancerous lesions such as oral leukoplakia [19] and oral lichen planus [20]. Thus, even though wound healing and cancer progression share regulation commonalities including proliferation and migration, our data should not be extrapolated towards physiological wound healing but used more as a basis to critically evaluate PRF therapy for oral cancerous lesions [49]. Importantly, we show here that PRF reduces the proliferation of oral epithelial cells, presumably because of its TGF-β content [50]. Thus, our findings do not provide a direct explanation of how PRF membranes support oral wound healing and prevent gingival recessions, a process that presumably involves the proliferation and migration of oral epithelial cells.

## 5. Conclusions and Future Research

Our data showing that PRF membranes support the proliferation and migration of HSC2 oral squamous carcinoma cells should be considered as a primer for upcoming research that is more sensitive to treatment with PRF in cases of oral carcinoma. Future research could build upon our RNAseq approach combined with the respective bioassays and develop a more clinically relevant assay for PRF. For instance, by embedding a tissue explant from OSCC and healthy gingiva into a PRP membrane, we can study the outgrowth and proliferation of the respective epithelial cells. The staining of proliferation markers such as Ki67 or PCNA in histological samples of OSCC and gingiva biopsies is also possible. Not only PRF lysates but also the respective lysates from buffy coat and platelet-poor plasma (PPP) are mitogenic and support the cell motility of HSC2 cells. This is interesting as PPP is, in particular, considered cell-free, suggesting that plasma components affect cell fate; for instance, the lipid fraction of PPP has anti-inflammatory activity [51]. Thus, the preclinical translation of our in vitro findings is worth consideration in future research.

## Figures and Tables

**Table 1:** Primer sequences.

Genes	Forward Sequence	Reverse Sequence
CDC45	GAAGCGCACACGGTTAGAA	GTTCACTCCCAGAGCCACTC
CyclinD1	TCGGTGTCCTACTTCAAATGTGT	GGGATGGTCTCCTTCATCTTAG
E2F1	GGACCTGGAAACTGACCATCAG	CAGTGAGGTCTCATAGCGTGAC
FAM111B	GGTTCTGGGGAAATCCAGTC	AAGATGGAGAAACAAGGATTGAA
HIST1H1A	GGCAAAGAAACCTGCTAAGGCTG	TAAGAGCTGCCAACGACACACC
HIST1H1D	GCTTATCACCAAGGCAGTGGCA	CCAGAGTACCTTTGCTCACCAAG
HIST1H2AJ	CGACAACAAGAAGACTCGCATCA	TGTGCGATGGTGACTTTGCCCA
IL1b	ATGATGGCTTATTACAGTGGCAA	GTCGGAGATTCGTAGCTGGA
Ki-67	ATAAACACCCCAACACACACAA	GCCACTTCTTCATTCCAGTTACA
MCM10	CATGAAGCCCAAGGATGG	GACCTTCTGAGGATGATCGATAG
PCNA	TGGAGAACTTGGAAATGGAAAC	GAACTGGTTCATTCATCTCTATGG
SULT1B1	CCAACTACAGTGATGGATCATAGC	GCGGAATTGAAGTGCAGTTTTGG

**Table 2:** RNAseq. HSC2 cells were incubated with and without PRF lysates for 24 h. The RNA was then subjected to RNAseq analysis. Genes are ranked by expression changes, with a cut-off of 10-fold.

Upregulated Genes		Downregulated Genes	
HIST1H1A	83.0	FOXA1	21.4
IL1B	59.5	KCNH4	19.6
E2F1	56.1	SAMD11	18.9
HIST1H1D	43.3	DPP7	18.7
SULT1B1	35.9	TCHHL1	18.4
HIST1H2AJ	33.7	IFI44L	16.9
XYLT1	24.7	RGS16	16.5
MMP9	24.7	ROM1	16.0
NAV3	23.6	SH2D3C	14.9
OTUB2	22.4	PIFO	14.3
ZNF367	21.6	BLNK	14.3
ORC1	21.3	CACNA1A	14.3
LRRC75B	20.2	KRTAP19-1	13.4
EXO1	19.3	ATOH8	12.5
CAMP	19.1	S1PR1	12.5
CDCA7	18.2	CAMK2A	12.0
CLCF1	16.8	C10orf99	11.8
CCNE2	16.8	TMPRSS11E	11.6
F2R	16.5	TTLL3	11.6
SLCO4A1	16.3	PDE3A	11.3
ETV5	16.3	TMEM86A	11.1
FAM72D	15.7	SMAD9	10.9
IL1R2	14.9	ZNF691	10.7
MCM10	14.8	RCAN2	10.7
RAET1L	14.6	ACAA2	10.7
HIST1H2BF	14.6	CCL26	10.7
HIST2H2AC	14.6	WDR93	10.7
FAM111B	13.6	RBBP8NL	10.7
IL1A	13.5	SLC41A2	10.7
GPSM3	13.5	TM7SF2	10.6
HIST1H4C	12.9	NUPR1	10.6
PLXNA4	12.8	GBP2	10.4
MAMDC2	12.3	PAPLN	10.1
CDH11	12.3	SERPINI1	10.1
SPRED1	12.3		
AMIGO2	12.2		
AREG	11.7		
VEPH1	11.5		
VAX1	11.2		
MYBPHL	11.2		
KLF14	11.2		
GLI2	11.2		
ANKRD44	11.2		
IL1RN	10.9		
CLMP	10.7		
APCDD1L	10.4		
CDC45	10.3		
GPR68	10.1		

## Data Availability

The original contributions presented in the study are included in the article. Further inquiries can be directed to the corresponding author.

## Supplementary Materials

The following supporting information can be downloaded at: https://www.mdpi.com/article/10.3390/dj11100242/s1, Figure S1: RT-PCR analysis of proliferation markers; Figure S2: RT-PCR analysis of IL1; Figure S3: 3H-thymidine incorporations and cell counting.
