# Supplementary figures and images for: PRF Lysates Enhance the Proliferation and Migration of Oral Squamous Carcinoma Cell Lines
Source: Dent J (Basel). 2023 Oct 19;11(10):242. doi: 10.3390/dj11100242 (PMC10605502; doi:10.3390/dj11100242)

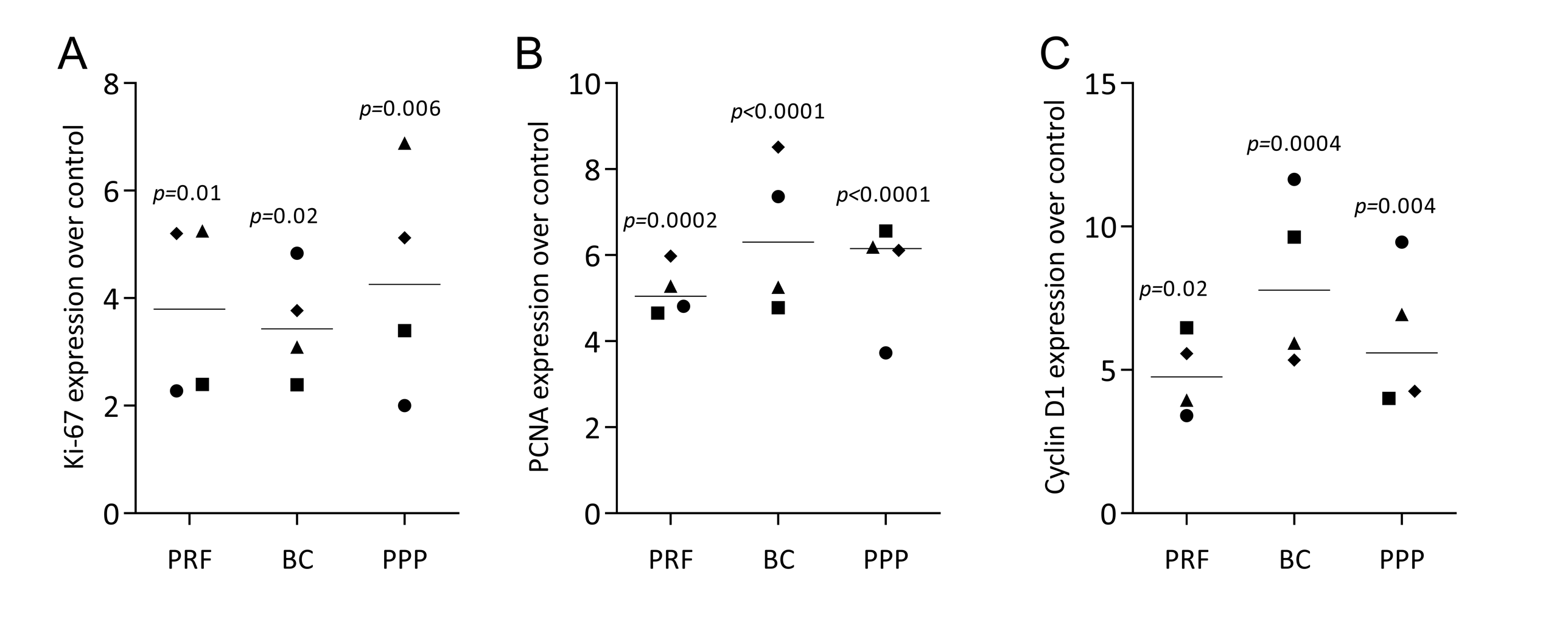

Supplement: Supplementary file 1 [file dentistry-11-00242-s001.zip › Supplement Figure 1.tif]

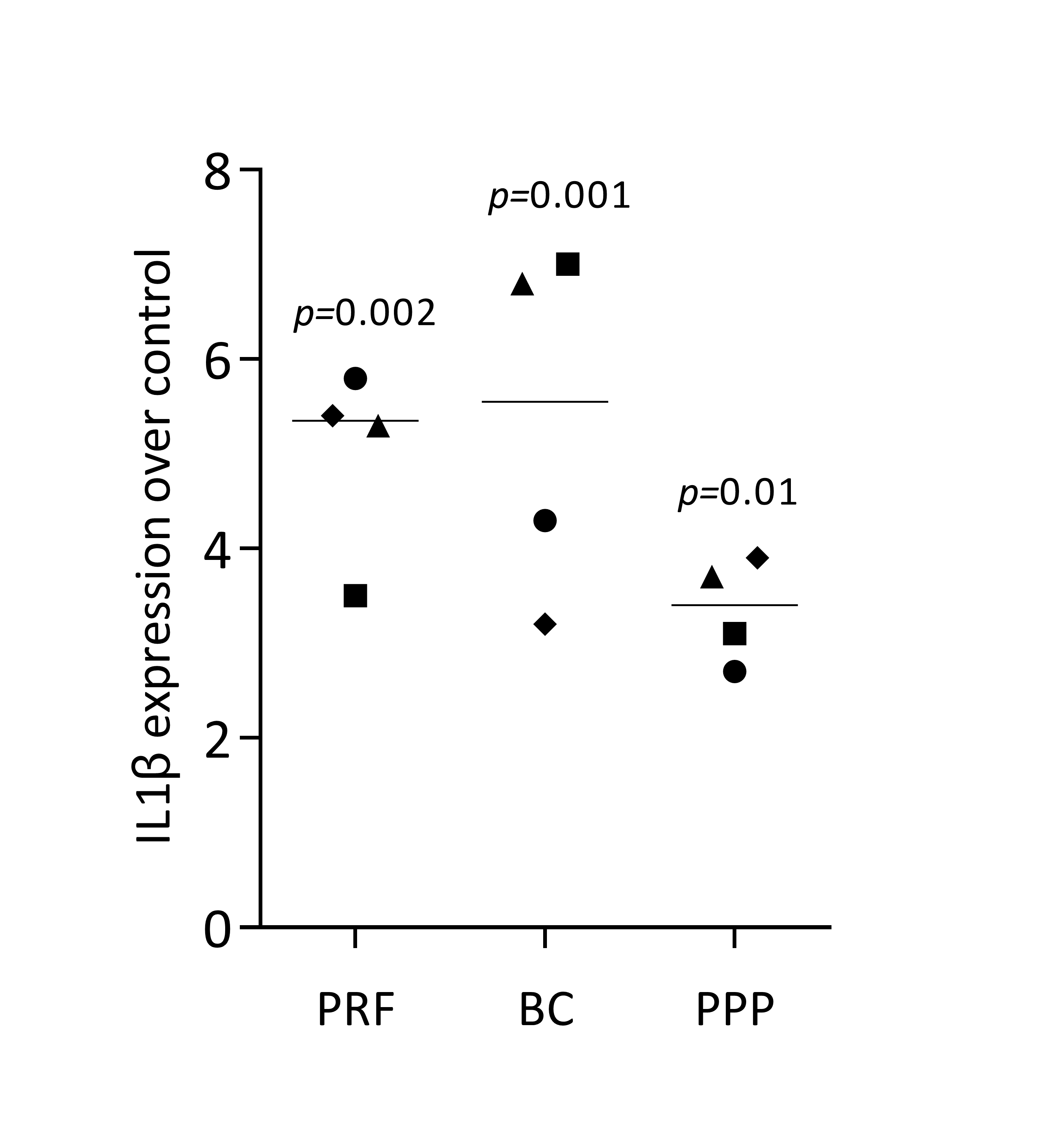

Supplement: Supplementary file 1 [file dentistry-11-00242-s001.zip › Supplement Figure 2.tif]

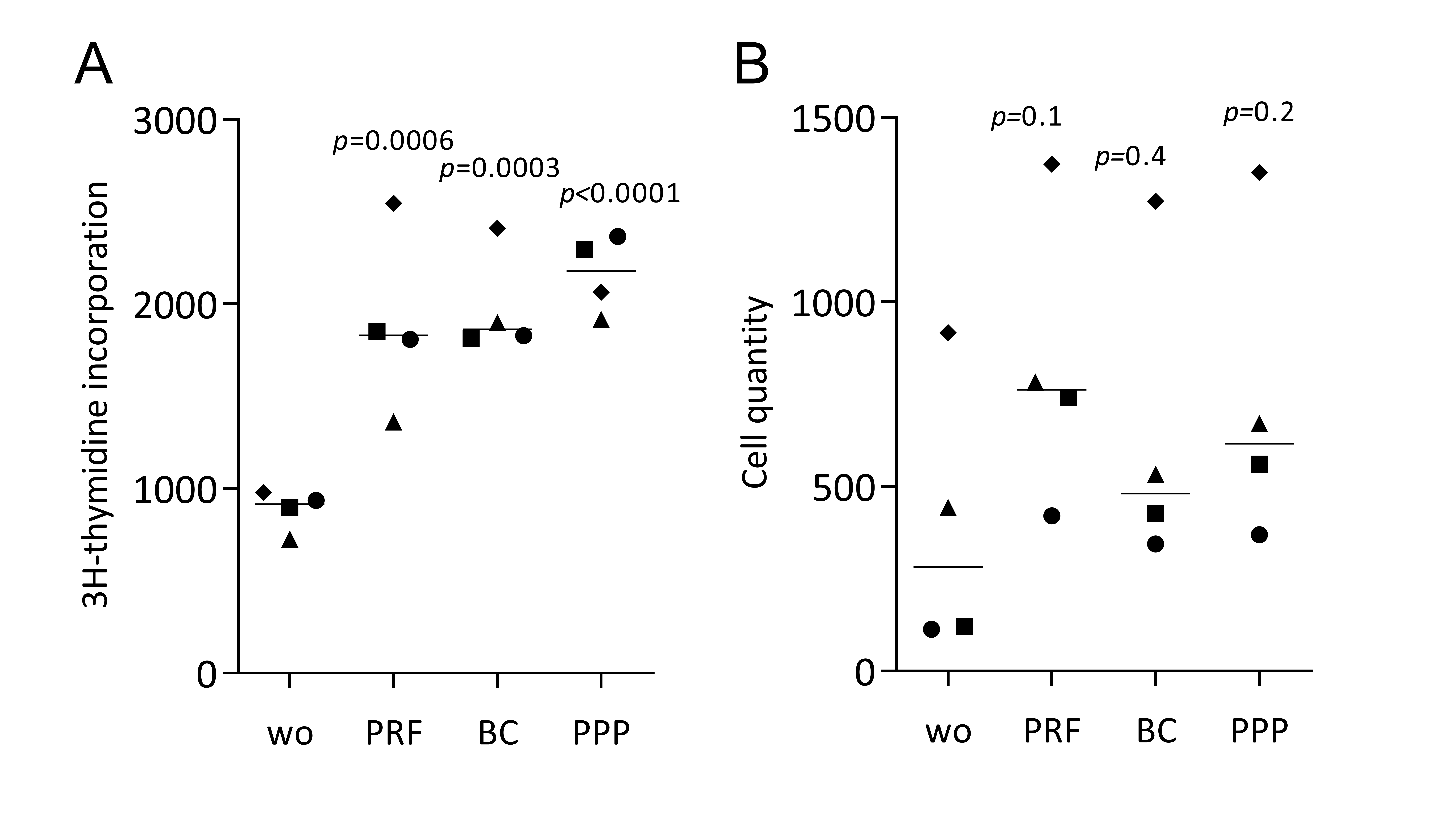

Supplement: Supplementary file 1 [file dentistry-11-00242-s001.zip › Supplement Figure 3.tif]
